# Nutritional Prehabilitation Intervention in Hematological Patients Undergoing Bone Marrow Transplant: A Systematic Review of the Literature

**DOI:** 10.3390/nu16244387

**Published:** 2024-12-20

**Authors:** Luca Falcone, Stefano Mancin, Elena Azzolini, Francesco Colotta, Sergio Ferrante, Manuela Pastore, Sara Morales Palomares, Diego Lopane, Marco Sguanci, Simone Cosmai, Daniela Cattani, Emanuele Cereda, Riccardo Caccialanza, Beatrice Mazzoleni

**Affiliations:** 1 Department of Biomedical Sciences, Humanitas University, Via Rita Levi Montalcini 4, Pieve Emanuele, 20090 Milan, Italy; luca.falcone@st.hunimed.eu (L.F.); stefano.mancin@humanitas.it (S.M.); elena.azzolini@humanitas.it (E.A.); francesco.colotta@humanitasresearch.it (F.C.); diego.lopane@hunimed.eu (D.L.); simone.cosmai@hunimed.eu (S.C.); daniela.cattani@humanitas.it (D.C.); beatrice.mazzoleni@hunimed.eu (B.M.); 2 IRCCS Humanitas Research Hospital, Via Manzoni 56, Rozzano, 20089 Milan, Italy; sergio.ferrante@grupposandonato.it (S.F.); manuela.pastore@humanitas.it (M.P.); 3 Department of Pharmacy, Health and Nutritional Sciences (DFSSN), University of Calabria, 87036 Rende, Italy; sara.morales@unical.it; 4 A.O. Polyclinic San Martino Hospital, Largo R. Benzi 10, 16132 Genova, Italy; marco.sguanci@unicampus.it; 5 Clinical Nutrition and Dietetics Unit, Fondazione IRCCS Policlinico San Matteo, 27100 Pavia, Italy; r.caccialanza@smatteo.pv.it

**Keywords:** bone marrow transplantation, nutrition, prehabilitation, systematic review

## Abstract

Background: Nutritional interventions play a critical role in bone marrow transplant (BMT) patients. This review evaluates the effectiveness of nutritional strategies in mitigating post-transplant malnutrition and improving clinical outcomes. Methods: A systematic review was conducted using PubMed, CINAHL, Cochrane Library, and Embase. The search terms included “bone marrow transplant”, “malnutrition”, and “preoperative nutritional interventions”. The quality of studies and risk of bias were assessed using the JBI framework, while evidence certainty was evaluated with the Oxford OCEBM. Results: Six studies were included (n = 3545 screened). The studies demonstrated predominantly high methodological quality and a low risk of bias, although heterogeneity in the treatments investigated and small sample sizes limited the evidence. Nutritional interventions significantly increased energy intake (26 vs. 24 kcal/kg/day, *p* = 0.038) and improved body weight (25% vs. 9%) with protein supplementation. Clinical complications decreased, including severe acute graft-versus-host disease (17.1% vs. 43.4%, *p* = 0.001) and pneumonia (27.6% vs. 52.7%, *p* = 0.002). The length of hospital stay (27 vs. 32 days, *p* = 0.006) and the need for parenteral nutrition (53% vs. 62%, *p* = 0.03) were also reduced. Overall survival improved with ≥50% adherence to prescribed TGF-beta2 intake (33 vs. 25.1 months, *p* = 0.03). Conclusions: Nutritional prehabilitation shows promise in improving BMT outcomes. Standardized nutritional programs could optimize care, although limitations in current evidence are clearly present. Larger randomized studies are needed to confirm findings and refine pre-transplant protocols.

## 1. Introduction

Cancer is one of the leading causes of mortality worldwide and remains a significant challenge in both the medical and healthcare fields. Hematological cancers account for approximately 6.5% of all cancer cases [1].

When conventional treatments prove ineffective or unsuitable, hematopoietic stem cell transplantation (HSCT) emerges as an advanced and complex therapeutic option for treating a variety of hematological malignancies, including leukemia, lymphoma, multiple myeloma, and myelodysplastic syndromes [2,3,4]. This procedure involves replacing diseased or damaged bone marrow with healthy stem cells capable of regenerating the hematopoietic system. By producing new blood cells, HSCT restores the patient’s immune function and bone marrow capacity, enabling optimal hematological and immunological recovery [5].

Two primary types of HSCT are recognized: autologous and allogeneic [2]. Each type has distinct characteristics and specific requirements, as differences in stem cell sources and procedural methods lead to variations in complication risks, recovery timelines, and preventive support strategies [6]. Autologous HSCT utilizes the patient’s own stem cells, which are collected, cryopreserved, and reinfused after a chemotherapy conditioning phase. This approach facilitates rapid hematological recovery, with primary risks stemming from the side effects of high-dose chemotherapy [7]. In contrast, allogeneic HSCT employs stem cells from a compatible donor, leveraging the donor’s immune system to target residual disease in the patient. This procedure aims to replace diseased marrow with healthy donor cells; however, it is associated with specific complications, such as graft-versus-host disease (GvHD). This necessitates prolonged monitoring and extended follow-up to manage and mitigate potential long-term complications [5,8].

**Figure 1:**
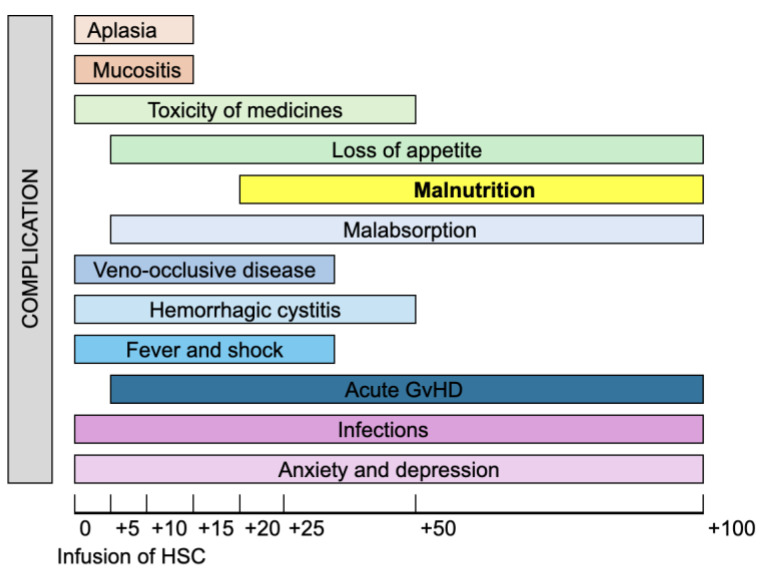
Time distribution of possible complications in the first 100 days after hematopoietic stem cell transplantation. Legend: HSC = hematopoietic stem cell.

It is important to emphasize that hematological patients undergoing these procedures are particularly vulnerable, primarily due to their immunocompromised status, resulting from both chemotherapy and the transplant itself [9]. High-dose chemotherapy regimens, essential for transplant success in both autologous and allogeneic procedures, significantly compromise the patient’s nutritional status [10]. This deterioration, combined with the inherent fragility of these patients, increases their susceptibility to post-transplant complications, lengthens hospital stays, and elevates mortality risk [11,12,13,14,15] (Figure 1).

Malnutrition in patients awaiting HSCT represents a critical clinical concern. It is defined as a condition resulting from inadequate nutrient intake or absorption, leading to alterations in body composition, cellular mass, and physical and mental function, with adverse effects on clinical outcomes [16]. Although many patients begin the transplantation process with an adequate nutritional status, a rapid decline is often observed in the weeks following the procedure. Studies report a significant increase in malnutrition prevalence, rising from 4–6% at admission to 35–60% at discharge [17,18]. Various side effects, such as loss of appetite, nausea, vomiting, diarrhea, and mucositis, are frequently observed, severely limiting oral intake [19]. Mucositis, which affects up to 75% of transplant patients, is particularly debilitating, causing painful symptoms that hinder food intake, compromise quality of life, and often result in significant weight loss—typically between 4% and 5% by discharge [20,21]. Additionally, GvHD, a complication specific to allogeneic transplantation, can further exacerbate malnutrition by frequently involving the gastrointestinal tract, leading to diarrhea, malabsorption, and reduced oral intake [17] (Table 1).

Despite the growing importance of nutritional prehabilitation interventions in cancer patients [22], there is limited evidence available for patients undergoing HSCT [15]. Specifically, while it is widely recognized that such interventions can improve clinical outcomes, the significant variability in methodologies and in the parameters measured across studies makes it challenging to definitively determine which nutritional interventions are most effective. Furthermore, the lack of standardized protocols for pre-transplant nutritional management and the differences in the criteria used to measure clinical outcomes complicate the ability to draw definitive conclusions. As a result, a systematic evaluation of the available evidence is needed to identify the optimal nutritional approaches for patients undergoing HSCT.

In response to these challenges, nutritional prehabilitation has emerged as a promising approach to counter the adverse effects of transplantation on nutritional status and patient frailty. This multidisciplinary intervention aims to optimize the patient’s nutritional status, enhancing their recovery capacity and reducing the risk of postoperative complications [23].

### Systematic Review Objectives

Given the impact of malnutrition and frailty on patients undergoing HSCT, along with the evidence for potential benefits of nutritional prehabilitation, the primary objective of this systematic review (SR) was to identify, through the existing literature, nutritional prehabilitation interventions and strategies aimed at preparing patients for the procedure. Specifically, this SR sought to determine the following, in order to reduce the incidence and severity of malnutrition and improve overall clinical conditions for patients: (1) the identification and effectiveness of current nutritional prehabilitation interventions and strategies in relation to nutritional status, biochemical parameters, and anthropometric measurements; (2) postoperative clinical outcomes, including postoperative complications and infections; (3) the healthcare professionals involved in nutritional prehabilitation processes along the patient care pathway.

## 2. Materials and Methods

### 2.1. Review Methodology

This SR was reported in accordance with the Preferred Reporting Items for Systematic Reviews and Meta-Analyses (PRISMA) guidelines [24] and following the PRISMA checklist (Supplementary File S1).

### 2.2. Systematic Review Protocol Registration

The protocol of this SR was registered in the International Prospective Register of Systematic Reviews (PROSPERO) of the National Institute of Health Research, available at https://www.crd.york.ac.uk/prospero/ with the protocol registration number CRD42024562143.

### 2.3. Formulation of the Research Question

The research question for this review was formulated using the PICO tool [25]. The PICO framework assists authors in constructing a focused research question for a review by addressing four main components: Population and Problem (P), Intervention of Interest (I), Comparison (C), and Outcome (O). For the purposes of this review, these four key aspects (PICO) were adapted as follows: P = patients with hematological disease who are candidates for bone marrow transplantation; I = nutritional prehabilitation interventions; C = standard of care or absence of nutritional prehabilitation interventions; O = effectiveness of nutritional prehabilitation in patients awaiting bone marrow transplantation, and its impact on postoperative outcomes, including identification of healthcare professionals involved in the prehabilitation phase.

### 2.4. Search Strategy

A comprehensive and systematic literature search was conducted between June and July 2024 to identify relevant and contemporary sources on nutritional prehabilitation interventions in the context of HSCT. Major scientific databases, including PubMed-Medline, Cochrane Library, CINAHL, and Embase, were thoroughly searched. To ensure an exhaustive and holistic analysis, hospital-specific databases and other repositories of gray literature were also explored, as these sources often provide valuable insights that are not covered in conventional scientific publications. The search strategy incorporated terms such as “bone marrow transplant”, “malnutrition”, and “nutritional prehabilitation intervention”, along with relevant synonyms and related phrases. Boolean operators (AND and OR) were applied thoughtfully to combine these terms, ensuring a broad yet focused search (Supplementary File S1).

During the initial screening phase, two researchers (L.F. and S.M.) independently reviewed all titles and abstracts retrieved from the database searches. Using EndNote 20^®^ software [26], duplicates and irrelevant records were systematically removed. In cases of disagreement, a third researcher (D.C.) was consulted to reach consensus. For studies deemed potentially relevant, the full articles were obtained and underwent rigorous independent assessment by two researchers (L.F. and S.M.), following predefined eligibility criteria. In situations where consensus was difficult to achieve, discussions among the primary reviewers were initiated. If no agreement could be reached, the opinion of the third researcher (D.C.), who had not been previously involved with the specific document, was sought to ensure an impartial decision-making process.

### 2.5. Criteria and Process

The inclusion criteria for this review encompassed quantitative primary studies published in English that investigated any form of nutritional prehabilitation intervention. These included educational, pharmacological, and non-pharmacological approaches initiated prior to conditioning regimens, with the goal of improving nutritional status and other clinically relevant outcomes, such as enhanced quality of life or reduced postoperative complications and follow-up issues. Eligible studies were required to involve adult patients with hematological diseases who were candidates for bone marrow transplantation.

Conversely, the exclusion criteria systematically excluded secondary studies, such as narrative and systematic reviews, qualitative studies, book chapters, articles without accessible full text, editorials, and publications of low methodological quality. Studies published in languages other than English, those involving pediatric patients or non-candidates for bone marrow transplantation, or those that did not include nutritional prehabilitation interventions were also excluded. Additionally, studies focusing solely on nutritional screening tools without implementing associated interventions were excluded. This rigorous selection process was implemented to ensure the scientific integrity, relevance, and applicability of the studies included in this systematic review.

### 2.6. Evaluation of Risk of Bias and Methodological Quality of Studies

The risk of bias and methodological quality of the included articles were assessed independently by two researchers (L.F. and S.M.). Any conflicts were resolved through consultation with a third researcher (D.C.). To ensure a rigorous evaluation of methodological quality and relevance, the Joanna Briggs Institute (JBI) Critical Appraisal Tools were employed [27]. These tools, renowned for their comprehensive approach to evaluating diverse research designs, provided a structured framework to assess the reliability and applicability of each study. High-quality studies were identified based on a classification system from a previous study [28]. Specifically, studies with a JBI score >70% were categorized as high quality, those scoring between 70% and 50% as medium quality, and studies with a score <50% as low quality (Supplementary File S1).

### 2.7. Assessment of Evidence Certainty

The certainty of evidence was evaluated using the framework established by the Oxford Centre for Evidence-Based Medicine (OCEBM) [29], which aligns more closely with the practical applications used by clinical researchers. This framework categorizes research into five distinct levels of evidence based on study design and research quality. High-level studies, including systematic reviews of randomized controlled trials (RCTs) and well-conducted individual RCTs, were classified as Level 1 evidence. Conversely, studies primarily based on expert consensus or lacking empirical support were assigned to Level 5. Intermediate-level research, such as less rigorous RCTs, single-arm trials, and methodologies including case series or case–control investigations, was classified as Levels 2, 3, and 4, respectively. In some cases, studies were re-evaluated and their evidence level adjusted—either elevated or downgraded—based on factors such as methodological rigor, precision of results, and relevance to the research topic [30].

### 2.8. Data Extraction

Data from the selected articles were extracted and summarized in tables, capturing the following information: authors, year of publication, country, study design, population, type of nutritional prehabilitation, and assessment of quality/bias.

### 2.9. Synthesis Methods

The articles included in this SR were systematically categorized based on nutritional prehabilitation interventions, optimal timing for their application, and the professionals involved. For each of these intervention classifications, the study methodologies and primary outcomes were articulated through a narrative synthesis. In this type of SR, the advantages of conducting a meta-analysis are widely recognized. However, a combined quantitative synthesis was deemed impracticable due to the heterogeneous nature of the included studies, as outlined in the *Cochrane Handbook for Systematic Reviews of Interventions* [31]. This heterogeneity was characterized by variations in the intervention types and methodologies used to quantify variable relationships, resulting in inconsistencies in both methodological and statistical aspects. As an alternative, a detailed narrative synthesis was conducted, adhering to the Synthesis Without Meta-analysis (SWiM) reporting guideline [32]. This approach was selected for its effectiveness in transparently and robustly synthesizing diverse quantitative data, while ensuring compliance with the PRISMA methodology.

## 3. Results

### 3.1. Search Results

**Figure 2:**
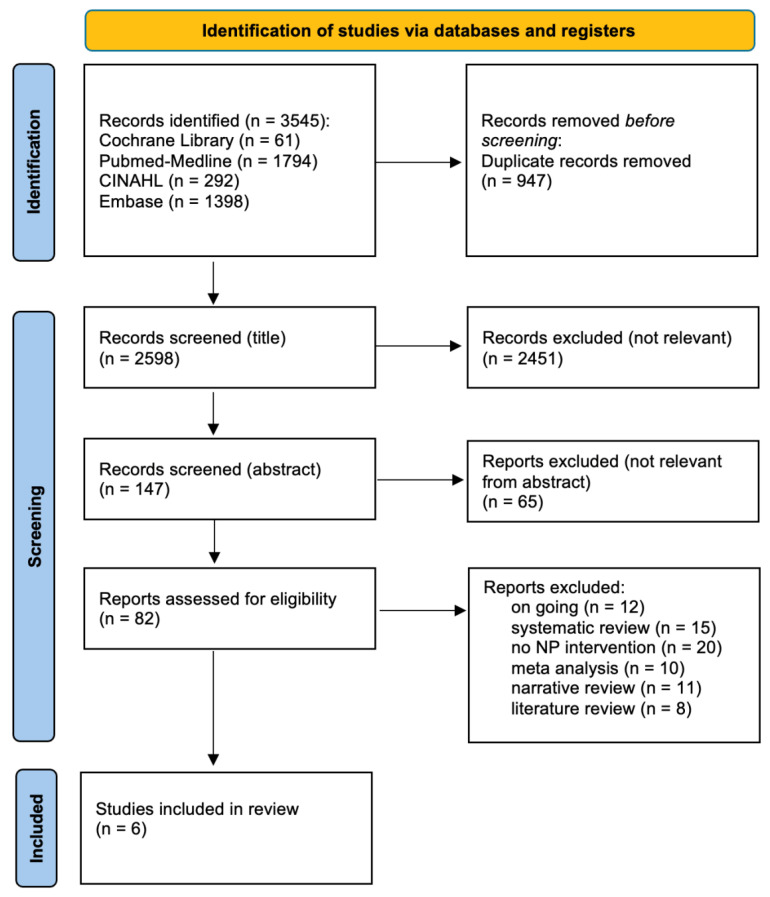
PRISMA flow diagram. Legend: NP = nutritional prehabilitation.

An extensive search process across various databases initially identified a total of 3545 records, distributed as follows: 61 from the Cochrane Library, 1794 from PubMed-Medline, 292 from CINAHL, and 1398 from Embase. The initial screening phase involved the removal of 947 duplicates, resulting in 2598 records for more detailed examination. A subsequent manual screening of titles excluded 2451 articles deemed irrelevant to this study, reducing the number of records to 147. These were then subjected to abstract-based screening, during which an additional 65 reports were excluded due to lack of relevance. As a result, 82 reports remained for eligibility assessment. After a thorough evaluation of these 82 reports, 76 were excluded for the following reasons: 12 were ongoing studies, 15 were systematic reviews, 20 did not present relevant interventions, 10 were meta-analyses, 11 were narrative reviews, and 8 were literature reviews. This rigorous screening process ultimately identified six studies that met the inclusion criteria. To provide a clear visual representation of the screening process and its outcomes, a PRISMA flow diagram is presented in Figure 2.

### 3.2. Characteristics of Studies, Populations, and Interventions

This SR included a heterogeneous collection of research methodologies encompassing various study designs. Specifically, five single-arm trials [33,34,35,36,37] and one RCT [38] were identified. The geographical distribution of these studies was predominantly concentrated in Asia, with three conducted in Japan [34,35,37] and one in China [38]. The remaining studies originated from European countries, including Italy [33] and Slovenia [36]. The total sample population across all studies consisted of 366 patients, with individual sample sizes ranging from 24 to 133 participants. Some studies employed both an intervention group (IG) and a control group (CG) [33,34,35,38]. Regarding the transplant types, four studies focused exclusively on allogeneic transplants [33,34,37,38], while two studies [35,36] included both allogeneic and autologous transplants. The interventions evaluated included a range of nutritional strategies: nutritional counseling [34,37], protein supplementation (PS) [38], specialized diets such as oral elemental diets [35], and oral nutritional supplements (ONSs) [36]. Additionally, one study [33] investigated the use of TGF-FSMP. These interventions primarily targeted three key phases of the transplant process: pre-transplant, post-transplant, and post-discharge. Most studies focused on the pre-transplant and post-transplant phases, with some [34,36,37] extending their interventions to the post-discharge phase. Conversely, other studies [33,38] did not specify details regarding post-discharge interventions. The duration and intensity of the interventions varied across studies, but a general trend emerged toward continuous nutritional support starting in the pre-transplant phase and extending into subsequent phases. This diversity in methodological approaches, types of interventions, and study populations provides a comprehensive perspective on current nutritional management strategies across different clinical and geographical contexts. Lastly, the included studies demonstrated predominantly high methodological quality and a low risk of bias. The methodological quality, assessed using the JBI checklists, showed an average score of 76.13%, with a range from 63.6% to 90.9%. The certainty of evidence, evaluated through the Oxford Centre for Evidence-Based Medicine (OCEBM) framework, was adequate across all studies. The evidence levels were distributed as follows: Level 2 for the study conducted by Ren et al. [38], and Level 3 for the remaining five studies [33,34,35,36,37] (Table 2).

#### Assessment of Nutritional Status

Assessing nutritional status in patients undergoing HSCT is critical for effective clinical management, but the methods vary across studies. Two studies [37,38] adopted a multiparametric approach, combining nutritional intake assessment, anthropometric measurements, and body composition analysis with biochemical parameters. Inden et al. [37] used InBody S10^®^ (TANITA, Tokyo, Japan) [39] to measure the skeletal muscle mass index (SMI), extracellular water/total body water (ECW/TBW) ratio, and phase angle (PA). They also integrated quality-of-life assessment using the EORTC QLQ-C30 questionnaire. Ren et al. [38] added muscle strength assessment through hand grip strength to their anthropometric and dietary evaluations.

In contrast to previous approaches, Morello et al. [33] distinguished themselves by using the PG-SGA, a standardized and validated tool for nutritional status assessment. Evaluations were conducted upon admission and then repeated at regular intervals during the post-transplant period, providing a longitudinal view of patients’ nutritional status. Unlike multiparametric approaches, the study by Rupnik et al. [36] opted for a more simplified approach, focusing on basic anthropometric measurements (weight, height, BMI), body composition analysis via bioimpedance, and dietary intake evaluation. They also considered symptoms that could affect nutritional status, such as poor appetite and recent weight loss. Similarly to Inden et al. [37], Aoyama et al. [34] emphasized body composition analysis, using bioelectrical impedance analysis (BIA) with the InBody S20^®^ tool (TANITA, Tokyo, Japan) [39]. They evaluated weight loss, skeletal muscle mass, and fat mass, along with calculating energy requirements and monitoring nutritional intake. They also included the assessment of nutrition-related adverse events.

Finally, Morishita et al. [35] chose an indirect approach to nutritional status assessment, using the duration of hospitalization as the primary endpoint. They also evaluated transplant-related complications and monitored food intake as indicators of patients’ nutritional status.

### 3.3. Interventions

**Figure 3:**
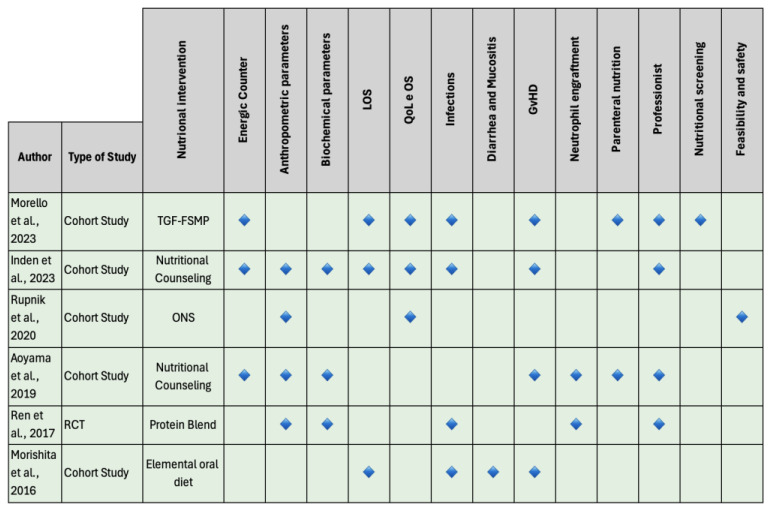
Nutritional interventions. Legend: Each row represents a different study, identified by the author and year of publication [33,34,35,36,37,38]. The columns indicate various aspects or parameters of the studies, such as the type of study, the nutritional intervention examined, and the different outcomes or measures considered. Blue diamonds indicate which parameters were analyzed in each study. LOS: length of stay; QoL: quality of life; OS: overall survival; GvHD: graft-versus-host disease; TGF-FSMP: transforming growth factor—Food for Special Medical Purposes; ONS: oral nutritional supplement; RCT: randomized controlled trial.

This SR identified a range of nutritional interventions across the included studies. All of the studies implemented personalized dietary plans, with some incorporating nutritional counseling and additional interventions (Figure 3).

Several studies utilized ONSs enriched with bioactive compounds. For instance, Morello et al. [33] administered a powdered food enriched with transforming growth factor beta 2 (TGF-beta 2), classified as a Food for Special Medical Purposes (FSMP). This product, initially developed for patients with inflammatory bowel disease (IBD), contains milk proteins, carbohydrates, lipids, vitamins, and minerals, along with TGF-beta 2, a cytokine known for its anti-inflammatory and immunomodulatory effects. The supplementation was tailored based on BMI and total daily energy expenditure (TDEE) and was initiated in the pre-treatment phase, continuing for 28 days post-transplant.

Parenteral nutrition (PN), following the EBMT guidelines, was provided to patients who declined the TGF-FSMP, ensuring adequate caloric and protein intake.

Similarly, in the study by Ren et al. [38], supplementation was given using a soy–whey protein blend: the product contained 50% whey protein and 50% soy protein. The rationale for combining these proteins is that whey protein is fast-digesting, causing a rapid amino acid peak in the blood, while soy protein is slower-digesting, providing a prolonged release of amino acids. The combination aimed to optimize amino acid availability over time, ensuring the stimulation of muscle protein synthesis, immune system support, and a potential positive effect on the gut microbiota. The administered dosage was 1.5 g/kg of body weight per day, from 30 days before the transplant to 30 days post-transplant. A 24 h food diary was used to monitor the patients’ daily energy and protein intake.

In contrast to previous approaches, the study by Morishita et al. [35] implemented an oral elemental diet: a specific nutritional approach composed of a liquid formulation of amino acids, easily digestible carbohydrates, minerals, and vitamins, with minimal fat content and fiber-free. This diet can be administered orally, avoiding the need for enteral or parenteral nutrition. The benefits of this composition include the intake of nutrients in an elemental or pre-digested form, a low osmotic load on the intestine, and easy absorption even in the presence of intestinal mucosal damage. It also helps reduce the digestive load on the intestine, providing essential nutrients in an easily assimilable form, potentially reducing intestinal inflammation. The dosage was 80 g/day, with a total treatment dose ranging from 2560 g to 2960 g, administered from the pre-treatment phase to 28 days post-transplant.

Finally, some studies adopted a broader approach based on personalized nutritional counseling combined with physical exercise, with the possible addition of oral nutritional supplements. For example, one study [36] included a protein powder supplement taken shortly after exercise, with a dosage of 0.3–0.4 g/kg of body weight per day, starting 2 weeks before the transplant, until the day of transplant. Two other studies [34,37] adopted a similar approach, aiming to achieve specific nutritional targets in terms of caloric and protein intake, with the addition of oral nutritional supplements (ONSs) for patients with a BMI below 20. Notably, the study by Inden et al. [37] aimed for a specific nutritional target of 31 kcal/kg/day and 1.0 g protein/kg/day; for patients with a BMI below 20, the study included supplementation with oral nutritional supplements providing 300 kcal and 20 g of protein. Similarly, another study [34] aimed for a specific target of 24–26 kcal per kg of ideal body weight per day and 0.7–0.9 g of protein per kg of ideal body weight per day. An interesting aspect of this study was the focus on energy balance, aiming to maintain an estimated basal energy expenditure (EBEE) between 104% and 115% of the estimated requirement, calculated using the Harris–Benedict formula.

### 3.4. Improvement in Nutritional Status

The nutritional status of the patients was assessed through the evaluation of energy intake, anthropometric parameters, and biochemical parameters (Table 3).

#### 3.4.1. Energy Intake and Malnutrition

Two studies evaluated the maintenance or improvement of energy intake. One study [34] reported a significant increase in total energy intake in the intervention group compared to the control group. Specifically, the intervention group achieved an energy intake of 26 kcal/kg of ideal body weight per day, compared to 24 kcal/kg in the control group (*p* = 0.038). Additionally, the study highlighted a higher adequacy rate for estimated basal energy expenditure (EBEE), calculated using the Harris–Benedict formula, in the intervention group. The EBEE adequacy reached 115% in the intervention group compared to 104% in the control group (*p* = 0.021).

Another study [33] demonstrated a significant reduction in the prevalence of severe malnutrition (PG-SGA C) in the intervention group compared to the control group. Specifically, the proportion of patients classified with severe malnutrition decreased to 28% in the intervention group, compared to 79% in the control group (OR 2.86, *p* = 0.001).

#### 3.4.2. Anthropometric Parameters

Four studies examined anthropometric parameters. Inden et al. [37] reported a statistically significant decrease in BMI (*p* < 0.001) starting 30 days post-transplant, with this trend persisting until discharge. This decrease was accompanied by significant reductions in triceps skinfold thickness (%TSF) at 60 days post-transplant and at discharge (*p* = 0.002), as well as in arm muscle circumference (%AMC) at discharge (*p* = 0.007). These changes occurred despite patients maintaining an energy intake of 31 kcal/kg/day and a protein intake of 1 g/kg/day.

Another study [36] demonstrated the effectiveness of a prehabilitation program in improving pre-transplant body composition. The patients exhibited a statistically significant increase in fat-free mass (FFM), rising from 56.7 ± 10.2 kg at the start of the program to 57.8 ± 9.5 kg at its conclusion, with an average increase of 1.1 kg (*p* = 0.011) over an average duration of 6.8 weeks.

Additionally, a study [34] highlighted the impact of advanced nutritional intervention in mitigating post-transplant losses in body weight (%LBW) and lean skeletal muscle mass (%LSMM). Specifically, the intervention eliminated differences in %LBW among patients with varying grades of intestinal GvHD (%LBW grade 0 = −1.7; grade 1–2 = −1.7; *p* = 0.98), a result not observed in the control group (%LBW grade 0 = −3.3; grade 1–3 = −9.0; *p* = 0.0006).

Ren et al. [38] further demonstrated that the supplemented group achieved better maintenance of or increases in several anthropometric parameters compared to the control group. Improvements included increases in body weight (25% vs. 9%), arm muscle circumference (75% vs. 17%), arm muscle area (66% vs. 25%), and calf circumference (58% vs. 25%). Additionally, the protein-supplemented group showed significantly greater improvements in grip strength (*p* < 0.050).

#### 3.4.3. Biochemical Parameters

Two studies examined serum albumin and total protein levels. One study [37] reported significantly lower albumin and total serum protein levels at 30 days post-transplant and at discharge compared to pre-transplant levels (*p* < 0.001). However, a trend toward recovery was observed by discharge.

Another study [38] demonstrated that 75% of patients in the protein supplementation group (BP) experienced a pre-transplant increase in albumin and total serum protein levels, compared to 25% in the control group (ND). Post-transplant, 58% of the intervention group maintained elevated levels, compared to 33% of the control group.

Conversely, Aoyama et al. [34] observed a median decrease in albumin of 0.7 g/dL in the control group and 1.0 g/dL in the nutritional intervention group on days 14–15 post-transplant (*p* < 0.0001). Additionally, the study noted a median increase in C-reactive protein (CRP) of 7.36 mg/dL in the control group and 7.17 mg/dL in the intervention group on day 12 post-transplant (*p* < 0.0001).

Regarding transthyretin (prealbumin), Inden et al. [37] reported that levels remained within the reference range throughout the observation period. Significant positive correlations were identified between pre-transplant transthyretin levels and quality-of-life scores at 60 days post-transplant. The same study also observed significantly lower zinc levels at 30 and 60 days post-transplant compared to pre-transplant levels (*p* = 0.006) (Table 4).

### 3.5. Length of Stay

The studies reviewed evaluated the impact of nutritional interventions on the length of hospital stay (LOS) in patients undergoing allogeneic hematopoietic stem cell transplantation.

Inden et al. [37] reported a median LOS of 97 days (range: 78–123 days) in patients who received early nutritional support. However, the study did not include a control group or perform a statistical analysis of LOS, limiting the ability to draw definitive conclusions about the effectiveness of the intervention. In contrast, Morello et al. [33] demonstrated a significant reduction in LOS with the use of a nutritional intervention based on TGF-FSMP. Patients in the intervention group, who consumed at least 50% of the prescribed TGF-FSMP dose, had a median LOS of 27 days, compared to 32 days in the control group consuming less than 50% (*p* = 0.006). This 5-day reduction was associated with other clinical benefits, including a lower prevalence of severe malnutrition (PG-SGA C), reduced reliance on PN, and fewer post-transplant complications. Similarly, Morishita et al. [35] found that patients in the intervention group had a significantly shorter median LOS compared to the control group (34 days vs. 50 days, *p* = 0.007), highlighting the positive impact of advanced nutritional interventions on recovery time (Table 5).

### 3.6. Quality of Life and Global Survival

Three studies reported on improvements in quality of life (QoL) and overall survival (OS). Inden et al. [37] evaluated the impact of early nutritional support on QoL using the EORTC QLQ-C30 questionnaire. Global health scores significantly decreased at 30 days post-transplant compared to pre-transplant levels but showed significant improvement between 30 and 60 days post-transplant (*p* < 0.001). Positive correlations were observed between pre-transplant transthyretin levels and QoL scores at 60 days post-transplant, including global health (r = 0.459, *p* = 0.027), physical functioning (r = 0.512, *p* = 0.012), cognitive functioning (r = 0.448, *p* = 0.032), and emotional functioning (r = 0.551, *p* = 0.006). These findings suggest that higher pre-transplant transthyretin levels predict better post-transplant QoL. The symptom scale scores for appetite loss, nausea/vomiting, fatigue, and diarrhea worsened significantly in the first 30 days post-transplant but improved between 30 and 60 days post-transplant and at discharge (*p* < 0.001). Pain and insomnia followed a similar pattern. Negative correlations were identified between symptoms such as fatigue (r = −0.511, *p* = 0.012) and pain (r = −0.544, *p* = 0.007) and QoL (*p* < 0.050). Similarly, another study [36] using the QLQ-C30 questionnaire reported improvements in QoL among participants in a nutritional program. Global health scores increased by 8.6 points (*p* = 0.006), emotional functioning by 8.3 points (*p* = 0.009), and social functioning by 12.1 points (*p* = 0.014). Reductions were noted in fatigue (13.4 points, *p* = 0.004), nausea (3.1 points, *p* = 0.042), and insomnia (10.8 points, *p* = 0.015).

Morello et al. [33] analyzed OS in patients receiving TGF-FSMP treatment. The intervention group, consuming ≥50% of the prescribed dose, had an estimated median OS of 33 months, significantly longer than the 25.1 months observed in the group consuming <50% (*p* = 0.03). While the relapse rates were not significantly different (21.1% in the intervention group vs. 24.1% in the control group), OS was positively influenced by TGF-FSMP intake ≥ 50% and negatively impacted by gastrointestinal GvHD, advanced disease at transplantation, and relapse. The study by Morishita et al. [35] found that non-relapse mortality at 100 days was lower, although not significantly (9.6% vs. 14.3%, *p* = 0.9) (Table 6).

### 3.7. Complications and Adverse Events

The analysis of adverse events and infectious complications in patients undergoing HSCT revealed some interesting findings regarding the impact of nutritional interventions.

#### 3.7.1. Infections

Regarding serious adverse events, the study by Morishita et al. [35] did not find statistically significant differences between the group receiving the elemental diet and the control group. Specifically, the incidence of documented infections was comparable between the two groups (7.7% vs. 4.8%, *p* = 0.67). Similarly, no significant differences were observed in the incidence of grade 2–4 fever according to the CTCAE v4.0 scale (83% vs. 67%, *p* = 0.13), nor in its mean duration (3.9 days vs. 5.0 days, *p* = 0.95).

However, the analysis of infectious complications, particularly pulmonary infections, revealed favorable outcomes associated with nutritional interventions. The study by Morello et al. [33] reported a significant reduction in the incidence of pneumonia in the group receiving TGF-beta supplementation compared to the control group (27.6% vs. 52.7%, *p* = 0.002). Similarly, Ren et al. [38] observed a lower incidence of pulmonary infections in the group receiving protein supplementation compared to the control group (41% vs. 66%), although no specific *p*-value was provided for this difference. Finally, Inden et al. [37] reported a 27% incidence of infections (seven patients) post-transplant (Table 7).

#### 3.7.2. Gastrointestinal Complications: Diarrhea and Mucositis

One study [35] provided data on the complication of mucositis, reporting promising trends in favor of nutritional intervention. Regarding the incidence of severe oral mucositis (grade 3–4), as assessed by the CTCAE v4.0 scale, a lower incidence was observed in the group receiving the elemental oral diet compared to the control group. Specifically, severe mucositis occurred in 25% of patients in the intervention group versus 48% in the control group. Although this difference was clinically relevant, it did not achieve statistical significance (*p* = 0.06).

Similarly, the analysis of the average duration of mucositis indicated an advantage for the elemental diet group. Patients receiving the elemental diet experienced a shorter average duration of mucositis (2.8 days) compared to the control group (5.6 days). However, this difference also failed to reach statistical significance (*p* = 0.07).

Unexpectedly, the study also revealed a tendentially higher incidence of grade 3–4 diarrhea in the intervention group compared to the control group, despite the protective intent of the nutritional intervention. Specifically, 50% of patients in the intervention group experienced severe diarrhea, compared to 26% in the control group. While this difference did not reach statistical significance (*p* = 0.08), it underscores the need for further investigation (Table 8).

#### 3.7.3. GvHD

The results of the studies reviewed here indicate a generally positive impact of nutritional prehabilitation interventions on the risk of complications in hematopoietic stem cell transplantation (HSCT), particularly regarding the incidence and severity of acute GvHD. The use of standardized tools for assessing GvHD enhanced the accuracy and comparability of results across studies.

Most studies employed a combination of clinical assessment and, when feasible, pathological confirmation to diagnose and classify GvHD. Morishita et al. [35], using the consensus criteria from the 1994 Conference on Acute GvHD Classification, observed a trend toward a lower incidence of acute GvHD grades II–IV at 100 days in the elemental diet group (15% vs. 30%, *p* = 0.26), although the difference was not statistically significant. Morello et al. [33], applying the MAGIC (Mount Sinai Acute GvHD International Consortium) criteria, reported a significant reduction in the incidence of acute gastrointestinal GvHD (9.2% vs. 34.5%, *p* = 0.001) and severe acute GvHD grades II–IV (17.1% vs. 43.4%, *p* = 0.001) in the group receiving nutritional supplementation.

In contrast, Aoyama et al. [34], using the Transplant Registry Unified Management Program (TRUMP) criteria, found no significant differences in the overall incidence of GvHD between groups. However, the improved nutrition group exhibited a significantly higher degree of cutaneous GvHD, with no differences observed in hepatic or gastrointestinal GvHD. Interestingly, in the control group, patients with gastrointestinal GvHD grade ≥1 experienced significantly greater body weight loss compared to those with grade 0 (−9.0% vs. −3.3%, *p* = 0.0006), a difference not observed in the intervention group. Finally, Inden et al. [37] reported a 38% incidence of acute GvHD grades II–IV, further underscoring the variability in outcomes across different nutritional interventions and assessment tools (Table 9).

#### 3.7.4. Neutrophil Enrichment

Two studies investigated the impact of nutritional interventions on engraftment, yielding partially contrasting results. Aoyama et al. [34] reported that neutrophil engraftment occurred significantly earlier in the control group compared to the nutritional intervention group (day +17 vs. day +19, *p* = 0.0438).

Conversely, Ren et al. [38] found that stem cell engraftment occurred significantly earlier in the group receiving protein supplementation compared to the control group. Specifically, the BP (protein supplementation) group achieved engraftment in 12.2 ± 2.0 days, whereas the ND (control) group required 15.1 ± 2.9 days (*p* < 0.050) (Table 10).

### 3.8. Duration of Artificial Nutrition

The impact of nutritional interventions on PN requirements and duration was investigated in two studies. Morello et al. [33] reported a significant reduction in PN use among patients with higher adherence to the nutritional intervention. Specifically, in the group consuming ≥50% of the prescribed TGF-FSMP dose, 67.5% of patients (52/77) avoided PN, compared to only 33.3% of patients (18/54) in the group consuming <50% of the prescribed dose (*p* = 0.000). Aoyama et al. [34] provided a detailed analysis of PN duration and characteristics across study groups. The mean PN duration was significantly shorter in the nutritional intervention group compared to the control group (53 days vs. 62 days, *p* = 0.03). However, no significant differences were found between the groups in terms of caloric intake (13 kcal/kg of ideal body weight/day in both groups, *p* = 0.86) or protein intake (0.5 vs. 0.4 g/kg of ideal body weight/day, *p* = 0.18) provided by PN. Similarly, the percentage of energy delivered through PN did not differ significantly between groups (51% vs. 57%, *p* = 0.19). Additionally, Aoyama et al. [34] observed a negative correlation between PN duration and oral energy intake in both groups (control group: r = −0.38, *p* = 0.009; nutritional intervention group: r = −0.37, *p* = 0.03). This finding suggests that higher oral energy intake is associated with reduced PN duration, regardless of the study group (Table 11).

### 3.9. Healthcare Professionals Involved

In research conducted on healthcare professionals involved in nutritional support for BMT patients, several specialized roles have emerged.

The NST, comprising a dietitian/nutritionist, physician, and specialized nurse, emerged as a fundamental component in the multidisciplinary approach to patients’ nutritional management. As highlighted by Inden et al. [37], the NST distinguished itself through early and proactive intervention, initiating support as soon as the transplant decision was made. This team conducted weekly meetings and regular monitoring rounds, carefully assessing patients’ nutritional status and food intake. Based on these assessments, the NST provided personalized recommendations, including dietary modifications, introduction of nutritional supplements, or PN implementation.

The roles of dietitians and nutritionists were emphasized in two distinct studies. Aoyama et al. [34] detailed the extensive responsibilities of the nutritionist, which range from pre-conditioning nutritional education to daily monitoring during hospitalization. This professional tracks patients’ weight, nutritional intake, and body composition, adjusting dietary plans based on individual preferences and treatment-related side effects. Additionally, they collaborate with the medical team to optimize PN when required. Ren et al. [38] further outlined the nutritionist’s responsibilities, which include designing personalized meal plans, assessing dietary intake through specialized questionnaires, administering protein supplements, and collecting detailed anthropometric data. Conversely, two studies, by Rupnik et al. [36] and Morishita et al. [35], described nutritional support interventions but did not specify the professional figures involved in their implementation.

## 4. Main Findings

This SR analyzed the effectiveness of nutritional prehabilitation interventions in patients undergoing HSCT. First, specific factors that have proven most effective in improving nutritional status before HSCT include protein supplementation, personalized nutritional counseling, and specialized diets such as ONSs and elemental diets. Two studies demonstrated the effectiveness of nutritional interventions. One study [36] showed a significant increase in fat-free mass (1.1 kg, *p* = 0.011). Ren et al. [38] reported greater improvements in body weight (25% vs. 9%), arm muscle circumference, and grip strength (*p* < 0.050) in the protein-supplemented group. Nutritional counseling has also been associated with increased caloric and protein intake, with energy intake reaching 31 kcal/kg/day and protein intake of 1.0 g/kg/day, particularly in patients with low BMI [37].

In terms of clinical outcomes, patients receiving nutritional prehabilitation showed significant improvements compared to those who did not receive such interventions. Nutritional interventions significantly reduced LOS. Morello et al. [33] reported a 5-day reduction in LOS (*p* = 0.006), while Morishita et al. [35] found a 16-day reduction (*p* = 0.007). Regarding the early initiation of nutritional interventions, this review found that early nutritional support significantly reduced the incidence of severe complications, including pneumonia (*p* = 0.002) [33] and severe acute GvHD (17.1% vs. 43.4%, *p* = 0.001) [33].

The type of nutritional supplement, whether liquid or solid, also influenced the efficacy of the interventions. Liquid elemental diets, which are easier to digest and absorb, were particularly beneficial for patients with swallowing difficulties, nausea, or appetite loss. These diets, consisting of amino acids and easily digestible carbohydrates, helped mitigate the adverse effects of mucositis and gastrointestinal side effects [35]. Furthermore, nutritional interventions played a crucial role in reducing the dependency on PN in post-transplant patients. The patients adhering to the prescribed nutritional interventions had a significantly lower need for PN (*p* = 0.000) [33], and the duration of PN was significantly shorter in the intervention group (*p* = 0.03) [34].

Finally, factors contributing to the heterogeneity observed in the studies included differences in the intensity and duration of the nutritional interventions, as well as variations in conditioning regimens. These differences in protocols and study designs led to variability in the effectiveness of the interventions. Some studies employed more intensive interventions with longer durations, while others focused on specific nutrients or dietary counseling, which may have influenced the clinical outcomes and the extent of malnutrition reduction [33,37].

## 5. Discussion

This SR underscores the diverse strategies employed across studies, reflecting the evolving landscape of prehabilitation interventions in the context of hematopoietic cell transplantation (HCT).

Nutritional prehabilitation is increasingly recognized as a cornerstone in optimizing outcomes for patients undergoing HCT, a complex procedure associated with significant morbidity and a high risk of complications. Pre-transplant optimization plays a critical role in enhancing patient recovery and minimizing post-transplant risks.

In response to these challenges, six studies examined the efficacy of various nutritional interventions, including protein supplementation, specialized diets, and personalized nutritional counseling [33,34,35,36,37,38]. Nutrition is particularly crucial before transplantation, especially in patients who have undergone intensive chemotherapy treatments [15]. These individuals often face significant nutritional challenges, such as loss of appetite, nausea, vomiting, and altered taste perception. Addressing these issues is essential as part of a comprehensive and targeted pre-transplant management strategy. As demonstrated in studies by Ren et al. [38] and Morishita et al. [35], oral nutritional supplements and specialized diets show potential in mitigating post-transplant complications and expediting recovery in HCT patients. These interventions not only address the immediate nutritional deficits but also contribute to improving overall patient outcomes by enhancing tolerance to treatment and reducing the risk of adverse effects.

The findings of this review highlight the significant benefits of nutritional prehabilitation, including improved nutritional status, reduced post-transplant complications, and enhanced quality of life. For instance, Inden et al. [37] demonstrated sustained energy and protein intake in patients receiving early nutritional support, while Aoyama et al. [34] reported significantly higher energy intake in the intervention group. Similarly, a previous study [40] comparing early nutritional intervention with routine care found that patients in the intervention group achieved superior caloric and protein intake and experienced a significant reduction in moderate complications (8.2% vs. 25.2%). However, it is also important to highlight the presence of differences in nutritional protocols across studies [33,34,35,36,37,38]. Specifically, several studies included protein supplements, showing significant improvements in strength and anthropometric parameters. This could suggest that protein supplementation plays a key role in maintaining muscle mass and improving strength, particularly useful during post-transplant recovery. However, another important aspect to consider is the duration and intensity of the nutritional intervention, which are determining factors in achieving these results. The positive effects observed in these studies may be attributed to the personalized nutritional approach and the adjustment of the intervention intensity to meet the specific needs of the patients, as well as differences in postoperative treatment and rehabilitation protocols [41].

The positive effects of nutritional prehabilitation extend to a reduction in LOS, as evidenced by two studies [33,35]. This reduction not only improves patient outcomes but also has potential cost-saving implications for healthcare systems. A prospective study conducted in Spain further demonstrated that specialized nutritional support initiated within the first five days of hospitalization in patients with disease-related malnutrition reduced their LOS by 8.83 days compared to later initiation (95% CI: 3.55–14.10) [42]. Additionally, an SR [43] confirmed the effectiveness of nutritional prehabilitation, alone or combined with physical exercise, in reducing LOS, although the review focused on patients undergoing colorectal surgery. However, the median LOS of 97 days reported by Inden et al. [37] is notably longer compared to the aforementioned studies. This discrepancy may be attributed to differences in patient characteristics, including diagnosis, age, and variations in nutritional intervention protocols or discharge criteria. Unfortunately, unlike the study by Morello et al. [33], which focused on patients with acute myeloid leukemia and myelodysplastic syndromes, Inden et al. [37] did not provide detailed information on patient diagnoses, limiting the ability to draw direct comparisons.

Moreover, variability in conditioning regimens, discharge criteria, and healthcare settings can significantly impact clinical outcomes. For instance, differences in conditioning regimens, including the intensity and type of chemotherapy or radiation therapy administered, can influence the degree of immunosuppression and the patient’s overall vulnerability, leading to variations in post-transplant complications and recovery times [44]. Similarly, institutional differences in discharge criteria may affect the timing and nature of post-discharge care, thereby shaping recovery trajectories and the need for continued nutritional support [45]. Further research is needed to explore these variables and their influence on the effectiveness of nutritional support, thereby improving result comparability. The studies by Inden et al. [37] and Rupnik et al. [36] highlight the positive impact of nutritional prehabilitation on QoL in patients undergoing HSCT. Both studies found significant improvements in QoL, which is an essential consideration in the overall treatment and recovery process for these patients. The improvement in QoL observed in these studies suggests that nutritional prehabilitation may not only address physical nutritional deficits but also enhance psychological well-being, energy levels, and overall functional status. In other studies, similar findings have been reported, reinforcing the importance of considering QoL as a key outcome in prehabilitation interventions. For example, research has shown that nutritional support can reduce fatigue, improve physical function, and promote emotional well-being, all of which contribute to a better QoL during recovery [46]. These improvements are particularly important in HSCT patients, who often face a prolonged recovery period and numerous physical and psychological challenges. Moreover, the use of nutritional interventions appears to address both the direct and indirect effects of malnutrition, which are often overlooked in traditional treatment protocols. By optimizing nutritional status prior to transplantation, patients may experience better tolerance to treatment, fewer complications, and faster recovery, all of which positively influence QoL outcomes. Furthermore, other clinical studies involving head and neck cancer have shown that multimodal interventions, such as nutritional counseling, oral supplements, and swallowing exercises, improved nutritional status and quality of life, while reducing the risk of malnutrition [46,47]. The analysis of post-transplant complications produced mixed but promising results. Morello et al. [33] and Ren et al. [38] observed a significant reduction in the incidence of pneumonia in the intervention group. Regarding GvHD, Morello et al. [33] found a significant reduction in the incidence of acute gastrointestinal GvHD and severe acute grades II–IV GvHD in the intervention group, and this appeared to be partially related to preoperative nutritional status. Similarly, another study [48] demonstrated that prehabilitation in patients undergoing cardiovascular procedures reduces postoperative complications and improves functional capacities. These findings suggest that nutritional prehabilitation interventions may have a protective effect against some of the most feared complications of HCT.

Nutritional prehabilitation interventions have demonstrated a positive impact on the duration and utilization of PN. One study [33] reported a significant reduction in PN use among patients with higher adherence to the intervention (67.5% vs. 33.3%, *p* = 0.000). Another study [34] observed a shorter mean PN duration in the intervention group (53 vs. 62 days, *p* = 0.03) and identified a negative correlation between PN duration and oral energy intake in both groups (*p* < 0.05), suggesting that higher oral intake reduces the need for PN. The duration of artificial nutrition was also positively influenced by nutritional prehabilitation interventions. A separate study [49] highlighted that proper assessment of clinical and nutritional parameters significantly improves the effectiveness of artificial nutrition, reducing reliance on complex and costly interventions such as PN. The same study identified prealbumin levels and comorbidities as key predictors of artificial nutrition outcomes, suggesting that more targeted nutritional management could further optimize artificial nutrition use. However, variability in albumin level results was noted among the analyzed studies. While two studies [37,38] reported increased albumin levels in the intervention groups, another study [34] observed a decrease in both groups, with a greater reduction in the nutritional intervention group. This apparent contradiction may be attributed to factors such as measurement timing and differences in nutritional interventions, patient characteristics, and individual inflammatory responses. An SR [20] emphasized that albumin, although widely used as a nutritional biomarker, can be influenced by factors beyond malnutrition, including inflammation, hydration, and liver function. In the context of allogeneic HSCT, systemic inflammation and frequent transfusions further limit albumin’s reliability as a nutritional indicator. The review underscored the absence of a single “gold standard” biomarker for nutritional assessment in HSCT patients, advocating for a multi-omics approach. This method, which combines various biomarkers with patient-reported outcomes, could provide a more accurate and holistic assessment of nutritional status, taking into account the metabolic and inflammatory complexity of the transplant process.

This SR underscores the critical need for a comprehensive and integrated multidisciplinary approach to nutritional prehabilitation. The reviewed studies highlight the essential contributions of various healthcare professionals, including nutritionists, dietitians, and nutritional support teams, in ensuring the success of these interventions. In many healthcare systems, dietitians often serve as the primary reference for modifying dietary intake. However, the role of nurses, particularly those specialized in nutrition, is equally critical, as demonstrated in previous studies [50,51]. Nurses play a pivotal role in monitoring nutritional status, educating patients, and managing nutritional interventions within the context of prehabilitation pathways. To improve patient adherence to nutritional interventions, it is essential to implement personalized care plans that consider individual preferences and challenges, supported by regular follow-up and encouragement from the healthcare team [52]. Additionally, leveraging tools such as mobile apps and telemedicine can facilitate real-time monitoring and feedback, thereby increasing patient engagement and adherence to nutritional recommendations [53,54]. Furthermore, the involvement of psychologists can help address any emotional or psychological barriers that patients might face, such as stress, depression, or anxiety, which often interfere with their ability to follow nutritional guidelines. This collaborative, multidisciplinary effort ensures that patients receive holistic and integrated care. By addressing not only medical needs but also nutritional and psychosocial well-being, such an approach optimizes overall health outcomes and increases the likelihood of transplant success [13,55,56].

A significant aspect that emerged is the pre-transplant malnutrition risk assessment, which was often not explicitly performed in the studies reviewed. Many patients were preemptively identified as being at high risk of malnutrition, largely due to the use of the NRS 2002 scale [57]. This tool includes a specific indicator that automatically flags HSCT candidate patients as high-risk for malnutrition. The presence of this “checkbox” reduces the need for further detailed assessments, as the score already denotes a high-risk status, and assuming that all patients are at high risk without an individual assessment may lead to less precise and personalized management. The NRS 2002 scale is a useful screening tool, but it may not capture the complexity of nutritional needs specific to each patient, especially considering the multifaceted nature of malnutrition in HSCT patients, which can involve not just nutritional intake but also factors like inflammation, comorbidities, and treatment-related complications. To standardize the evaluation of preoperative malnutrition risk in HSCT patients and facilitate comparisons across studies, it is crucial to combine the NRS 2002 scale with additional clinical and biochemical measures, such as body composition analysis, serum albumin levels, and muscle mass evaluation [58]. Incorporating multidimensional approaches, including patient-reported outcomes (PROs), would personalize the assessment by capturing subjective factors like fatigue, appetite loss, and overall well-being [59]. This combination of objective and subjective assessments would provide a more comprehensive evaluation of nutritional status, enabling more accurate comparisons across studies and ensuring tailored nutritional management to optimize patient outcomes. Personalized evaluations not only ensure accurate risk identification but also facilitate the implementation of more targeted nutritional strategies, which can significantly improve transplant outcomes [60]. Furthermore, greater standardization and awareness in the application of malnutrition risk assessment scales within prehabilitation pathways are needed to optimize targeted nutritional interventions. Nonetheless, challenges persist in determining the precise timing, dosage, composition, and adherence to nutritional interventions. Prehabilitation pathways often exhibit heterogeneity, with variations in dosage, initiation timing, and intervention duration. The effectiveness of these prolonged nutritional interventions remains uncertain, primarily due to small or heterogeneous sample sizes and differences in the outcomes analyzed across the included studies.

### 5.1. Future Perspectives

To enhance the quality of studies in the field of nutritional prehabilitation for HSCT patients, future research should focus on standardizing methodologies, including intervention protocols, outcome measures, and assessment tools. Consistent use of validated measurement instruments and clearly defined treatment protocols will help produce more reliable and comparable results. Additionally, the inclusion of larger, multicenter RCTs would strengthen the generalizability of findings and reduce bias. Future studies should particularly investigate the specific needs of patients undergoing different types of transplantation, such as autologous, allogeneic, and chimeric antigen receptor T-cell therapy (CAR-T). Each of these transplant approaches presents unique challenges related to immunosuppression, GvHD, and recovery, which can impact nutritional status and post-transplant complications. Understanding how nutritional interventions can be optimized for each type of transplant will help identify the most effective strategies at various stages of the treatment process, ultimately improving patient outcomes.

### 5.2. Limitations

Despite the promising results, it is important to acknowledge some limitations of this review. The limited number of studies—the inevitable counterpart of a rigorous methodological approach—and the relatively small sample sizes in some of the included research raise concerns about the generalizability and potential bias of the results. Moreover, variability in clinical–nutritional pathways and protocols, along with differences in the duration, intensity, and specific approaches of nutritional interventions, introduces significant heterogeneity. These variations impact the consistency of outcomes, making it difficult to draw definitive conclusions across studies. Standardizing these pathways and protocols could help reduce heterogeneity, enhance the comparability of results, and provide more reliable evidence for optimal nutritional management in HSCT patients. Additionally, variations in study design and methodology, including differences in patient populations, outcome measures, and assessment tools, may impact the overall quality and reliability of the evidence. Such methodological inconsistencies highlight the need for more standardized and robust study designs to enhance the comparability and reproducibility of findings in this field.

## 6. Conclusions

This SR highlights the potential benefits of nutritional prehabilitation in improving outcomes for patients undergoing HSCT. Nutritional interventions, including counseling, protein supplementation, and specialized diets, demonstrated significant positive impacts on nutritional status, inflammatory response, QoL, hospital LOS, and post-transplant complications. The evidence also suggests that these interventions may reduce the incidence of severe complications such as pneumonia and GvHD, as well as minimizing reliance on PN.

Despite these promising findings, the significant heterogeneity in intervention types, methodologies, and patient populations across studies limits the generalizability of the results. Differences in methodologies for assessing nutritional status and intervention effectiveness highlight the need for a personalized approach that considers specific risk factors such as pre-existing malnutrition and comorbidities. Continuous nutritional assessment throughout all phases of transplantation could further optimize outcomes. A multidisciplinary approach involving nutritionists, dietitians, and other healthcare specialists is essential to ensure personalized and continuous support. Despite promising results, further studies, particularly large-scale RCTs with standardized protocols, are needed to validate and refine nutritional prehabilitation strategies.

Future research should focus on standardizing protocols, analyzing the cost-effectiveness, and evaluating the long-term efficacy of these strategies. Thoroughly assessing the risk of malnutrition in each patient, even using scales like the NRS 2002, would enable more targeted interventions and avoid ineffective standardized approaches. Integrating nutritional prehabilitation programs into the management of HSCT candidates could represent a significant step forward in improving clinical outcomes and quality of life for this vulnerable population.

## Figures and Tables

**Table 1:** Factors contributing to malnutrition in HSCT patients.

	Malnutrition Factor	Description
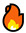	Inflammation	Infections, GvHD, post-transplant complications, treatment with chemotherapeutic drugs, and high-dose chemotherapy. Chronic inflammation can reduce nutrient absorption.
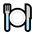	Reduced food intake	Nausea, vomiting, mucositis, diarrhea, and loss of appetite are common after the transplant, significantly reducing caloric intake.
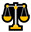	Unintentional weight loss	Weight loss is a key sign of malnutrition and can be aggravated by gastrointestinal side effects like mucositis and GvHD.
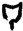	Altered intestinal absorption	Gastrointestinal complications can impair nutrient absorption.

Legend: HSCT: hematopoietic stem cell transplantation; GvHD: graft-versus-host disease.

**Table 2:** General characteristics of the included studies.

Author	Country	Design	Population (n)	BMT	Nutritional Assessment	Nutritional Intervention	Nutrition Characteristics (EN)	Limitations	OCEBM Level	Quality/Bias
Morello et al., 2023 [33]	Italy	Single-arm trial	(n = 133). IG = 76 CG = 53	Allogeneic	PG-SGA, anthropometric measurements, BMI, TDEE	TGF-FSMP ≥ 50% prescribed dose (IG) TGF-FSMP < 50% prescribed dose (CG)	TGF-FSMP dosage based on BMI and TDEE	Need for further studies to confirm the results	3	+++/Low
Inden et al., 2022 [37]	Japan	Single-armtrial	(n = 26)	Allogeneic	BIA (InBody S10)^®^, nutritional intake, anthropometric measurements, body composition, biochemical parameters, quality of life	Nutritional counseling (IG)	Nutritional education, evaluation of nutritional status/requirements. Kcal 31/kg/day. Protein 1.0 g/kg/day	Small sample size	3	++/Moderate
Rupnik et al., 2020 [36]	Slovenia	Single-arm trial	(n = 28)	All	Anthropometric measurements, bioimpedance, dietary intake assessment	ONS (IG)	Protein supplement (0.3–0.4 g/kg/day)	Lack of randomized control group; small sample size	3	+++/Low
Aoyama et al., 2019 [34]	Japan	Single-arm trial	(n = 82) IG = 36 CG = 46	Allogeneic	BIA (InBody S20)^®^, weight loss, muscle mass, fat mass, energy requirements, nutritional intake, adverse events	Nutritional counseling NSP (IG) SD (CG)	Nutritional education, evaluation of nutritional status/requirements. Kcal 24–26/kg/day. Protein 0.7–0.9 g/kg/day	Retrospective study without randomized groups	3	+++/Low
Ren et al., 2017 [38]	China	RCT	(n = 24) IG = 12 CG = 12	Allogeneic	Multiparametric approach: anthropometric measurements, muscle strength, biochemical parameters, dietary intake monitoring	PB (IG) SD (CG)	Protein supplement: 50% whey protein, 50% soy protein, protein blend 1.5 g/kg/day	Small sample size; short study duration; single-center study	2	+++/Low
Morishita et al., 2016 [35]	Japan	Single-arm trial	(n = 73) IG = 52 CG = 21	All	Not specified	ED (IG) SD (CG)	Amino acids (glutamine, arginine, BCAA), carbohydrates (79.3% dextrin), fats (0.6% soybean oil), minerals (2%), vitamins 80 g/day, total dose: 2560–2960 g	Single-center study; small sample size	3	++/Moderate

Legend: NSP = nutritional support pathway; SD = standard diet; PB = protein blend; ED = elemental diet; PS = protein supplement; ONS = oral nutritional supplement; RCT = randomized controlled trial; IG = intervention group; CG = control group; TDEE = total daily energy expenditure; BMT = bone marrow transplant; QoL = quality of life. Quality rating by JBI score [27]: +++, > 70% (high quality); ++, between 70% and 50% (medium quality).

**Table 3:** Anthropometric parameters.

Study	Parameters	Results	*p*-Value
Inden et al. (2022) [37]	BMI	Significant decrease at 30 days post-transplantation and at discharge	<0.001
	% TSF	Significant decrease at 60 days post-transplant and at discharge	0.002
	% AMC	Significant decrease at discharge	0.007
Rupnik et al. (2020) [36]	FFM	Increase from 56.7 ± 10.2 kg to 57.8 ± 9.5 kg (average increase of 1.1 kg)	0.011
Aoyama et al. (2019) [34]	% LBW	Significant mitigation in the intervention group	NR
	% LSMM	Significant mitigation in the intervention group	NR
Ren et al. (2017) [38]	Body weight	Increase in 25% of intervention group vs. 9% of control group	NR
	% AMC	Increase in 75% of intervention group vs. 17% of control group	NR
	Arm muscle area	Increase in 66% of intervention group vs. 25% of control group	NR
	Calf circumference	Increase in 58% of intervention group vs. 25% of control group	NR
	Grip strength	Significantly greater improvement in the intervention group	<0.050

Legend: AMC: arm muscle circumference; TSF: triceps skinfold thickness; FFM: fat-free mass; LBW: body weight loss; LSMM: skeletal muscle mass loss; NR: not reported.

**Table 4:** Biochemical parameters.

Study	Parameters	Intervention Group	Control Group	*p*-Value
Inden et al. (2023) [37]	Albumin and total protein	Significantly lower at 30 days post-transplantation and at discharge compared with pre-transplantation	NR	<0.001
Ren et al. (2017) [38]	Albumin and total protein (pre-transplant)	75% of patients with increased levels	25% of patients with increased level	NR
	Albumin and total protein (post-transplant)	58% of patients with increased levels	33% of patients with increased levels	NR
Aoyama et al. (2019) [34]	Albumin (days 14–15 post-transplant)	Median decrease of 1.0 g/dL	Median decrease of 0.7 g/dL	<0.001
	C-reactive protein (day 12 post-transplant)	Median increase of 7.17 mg/dL	Median increase of 7.36 mg/dL	<0.001
Inden et al. (2023) [37]	Transthyretin (prealbumin)	Maintained in the reference range throughout the period	NR	NR
	Zinc	Significantly lower levels at 30 and 60 days post-transplantation compared with pre-transplantation	NR	0.006

Legend: NR: not reported.

**Table 5:** Length of hospital stay.

Study	Intervention Group	Control Group	Difference	*p*-Value
Inden et al. (2023) [37]	97 days (median)	NR	Not applicable	NR
Morello et al. (2023) [33]	27 days (median)	32 days (median)	5 days	0.006
Morishita et al. (2016) [35]	34 days (median)	50 days (median)	16 days	0.007

Legend: NR: not reported.

**Table 6:** Quality of life.

Study	Outcome	Results	*p*-Value
Inden et al. (2023) [37]	QoL (overall health status)	Significant improvement between 30 and 60 days post-transplantation	<0.001
	Pre-transplant transthyretin correlation with QoL at 60 days	Overall health: r = 0.459	0.027
		Physical functioning: r = 0.512	0.012
		Cognitive functioning: r = 0.448	0.032
		Emotional functioning: r = 0.551	0.006
	Symptoms (appetite, nausea, vomiting, fatigue, diarrhea)	Worsening at 30 days, improvement between 30 and 60 days	<0.001
Rupnik et al. [36]	QoL (overall health status)	Improvement by 8.6 points	<0.006
	QoL (emotional functioning)	Improvement by 8.3 points	0.009
	QoL (social functioning)	Improvement by 12.1 points	0.014
	Fatigue	Reduction by 13.4 points	0.004
	Nausea	Reduction by 3.1 points	0.042
	Insomnia	Reduction by 10.8 points	0.015
Morello et al. (2023) [33]	Median OS (TGF-FSMP ≥ 50%)	33 months	0.03
	Median OS (TGF-FSMP < 50%)	25.1 months	-
	Incidence of recidivism (intervention group)	21.1%	NS
	Incidence of recidivism (control group)	24.1%	-
Morishita et al. (2016) [35]	Mortality unrelated to recidivism at 100 days (intervention group)	9.6%	0.900 (NS)
	Mortality unrelated to recidivism at 100 days (control group)	14.3%	-

Legend: QoL: quality of life; OS: overall survival; TGF-FSMP: transforming growth factor—Food for Special Medical Purposes; NS: not significant.

**Table 7:** Infections.

Study	Intervention Group	Control Group	Type of Infection	*p*-Value
Morishita et al. (2016) [35]	7.7%	4.8%	Documented infections	0.670
Morello et al. (2023) [33]	27.6%	52.7%	Pneumonia	0.002
Ren et al. (2017) [38]	41%	66%	Pulmonary infections	NR
Inden et al. (2023) [37]	27%	NR	Post-transplant infections	NR

Legend: NR: not reported.

**Table 8:** Gastrointestinal complications: diarrhea and mucositis.

Outcome	ED Group	Control Group	*p*-Value
Incidence of severe oral mucositis (grade 3–4)	25%	48%	0.06
Average duration of mucositis	2.8 days	5.6 days	0.07
Incidence of severe diarrhea (grade 3–4)	50%	26%	0.08

Legend: ED = elemental diet; data from the study by Morishita et al. [35].

**Table 9:** GvHD.

Study	Outcome	Intervention Group	Control Group	*p*-Value
Morishita et al. (2016) [35]	Acute GvHD grades II–IV at 100 days	15%	30%	0.260
Morello et al. (2023) [33]	Acute gastrointestinal GvHD	9.2%	34.5%	0.001
	Acute severe GvHD grades II–IV	17.1%	43.4%	0.001
Aoyama et al. (2019) [34]	Overall incidence of GvHD	ND	ND	NR
	Degree of cutaneous GvHD	Significantly higher	-	NR
	Degree of hepatic or gastrointestinal GvHD	ND	ND	NR
Inden et al. (2023) [37]	Acute GvHD grades II–IV	38%	NR	NR

Legend: NR: not reported; ND: no difference; GvHD: graft-versus-host disease.

**Table 10:** Neutrophil enrichment.

Study	Intervention Group	Control Group	Difference	*p*-Value
Aoyama et al. (2019) [34]	Day +19	Day +17	2 days later in the intervention group	0.04
Ren et al. (2017) [38]	12.2 ± 2.0 days	15.1 ± 2.9 days	2.9 days earlier in the intervention group	<0.05

**Table 11:** Artificial nutrition.

Study	Outcome	Intervention Group	Control Group	*p*-Value
Morello et al. (2023) [33]	Patients who avoided PN (≥50% TGF-FSMP dose).	67.5% (52/77)	-	0.00
	Patients who avoided PN (<50% TGF-FSMP dose)	33.3% (18/54)	-	0.00
Aoyama et al. (2019) [34]	Average PN duration	53 days	62 days	0.04
	Calorie intake from the PN	13 kcal/kg ideal weight/day	13 kcal/kg ideal weight/day	0.86
	Protein intake from the PN	0.5 g/kg ideal weight/day	0.4 g/kg ideal weight/day	0.19
	Percentage of energy supplied by PN	0.51	0.57	0.20

Legend: PN: parenteral nutrition; TGF-FSMP: transforming growth factor—Food for Special Medical Purposes; kcal/kg: kilocalories per kilogram.

## Data Availability

All available data are provided as Supplementary Files.

## Supplementary Materials

The following supporting information can be downloaded at: https://www.mdpi.com/article/10.3390/nu16244387/s1, Supplementary File S1: Search strategy; quality and risk of bias.
